# The role of carotid artery stenosis in predicting stroke after coronary artery bypass grafting in a Chinese cohort study

**DOI:** 10.1038/s41598-023-47640-5

**Published:** 2023-12-06

**Authors:** Shanghao Chen, Chuanxiao Mi, Shijie Zhang, Yi Li, Yan Yun, Xiangxi Zhang, Jianguang Chen, Yang Li, Haizhou Zhang, Tian Gao, Chengwei Zou, Xiaochun Ma

**Affiliations:** 1 Department of Cardiovascular Surgery, Shandong Provincial Hospital, Cheeloo College of Medicine, Shandong University, No. 324 Jingwu Road, Jinan, 250021 Shandong Province China; 2 Department of Cardiovascular Surgery, Shandong Provincial Hospital Affiliated to Shandong First Medical University, No. 324 Jingwu Road, Jinan, 250021 Shandong Province China; 3 Department of Radiology, Qilu Hospital of Shandong University, No. 107 West Wenhua Road, Jinan, 250012 Shandong Province China; 4 Dongying People’s Hospital, Dongying, Shandong Province China; 5 Department of Stomatology, Shandong Provincial Hospital Affiliated to Shandong First Medical University, No. 324 Jingwu Road, Jinan, 250021 Shandong Province China; 6 College of Pharmacy, Shandong University of Traditional Chinese Medicine, Jinan, 250355 Shandong Province China

**Keywords:** Cardiology, Health care, Risk factors

## Abstract

Current guidelines give priority to surgical treatment of carotid artery stenosis (CAS) before coronary artery bypass grafting (CABG), especially in symptomatic patients. Carotid artery stenting is an alternative treatment for narrowing of the carotid arteries. This study sought to demonstrate the role of severe CAS in predicting stroke after CABG and assess the efficacy of carotid artery stenting in preventing postoperative stroke in a Chinese cohort. From 2015 to 2021, 1799 consecutive patients undergoing isolated CABG surgery were retrospectively recruited in a Chinese cohort. The predictive value of severe CAS in postoperative stroke and carotid stenting in preventing postoperative stroke was statistically analyzed. The incidence of postoperative stroke was 1.67%. The incidence of CAS with stenosis ≥ 50% and ≥ 70% was 19.2% and 6.9%. After propensity matching, the incidence of stroke was 8.0% in the severe CAS group and 0% in the non-severe CAS group. We successfully established an optimal predictive nomogram for predicting severe CAS in patients undergoing CABG. Carotid artery stenting was found ineffective in preventing postoperative stroke. The present study provides the incidence of CAS and postoperative stroke in a Chinese cohort, identifies severe CAS as an independent risk factor for postoperative stroke after CABG, constructs a nomogram predicting the incidence of severe CAS, and evaluates the effectiveness of carotid artery stenting in preventing postoperative stroke after CABG.

## Introduction

Notwithstanding recent advances in coronary revascularization techniques and drug treatments, coronary artery disease (CAD) remains one of the most prevalent causes of death worldwide^1^. Coronary artery bypass grafting (CABG) has become the standard-of-care for patients with complex CAD due to its long-term reliability and effectiveness^2–4^. Data from previous large retrospective reports indicated that the overall incidence of perioperative stroke has decreased to 1.6%, and stroke remains one of the most common complications of CABG^5,6^. A previous study reported that approximately 40% of strokes occur intraoperatively and 58% postoperatively, with a peak at 40 h postoperatively^6^. Several studies reported that patients with stroke after CABG have more severe hospital complications, longer time in the ICU, longer postoperative stay, and higher in-hospital mortality^5,6^.

A few studies have shown that patients with carotid artery stenosis (CAS) have an increased risk of stroke after CABG^6–10^. However, controversy remains as to whether unilateral asymptomatic carotid stenosis is an independent risk factor for ipsilateral ischemic stroke after CABG^11–13^. In addition, carotid revascularization by concurrent carotid endarterectomy or carotid stenting appeared to be beneficial in patients with symptomatic carotid artery disease and bilateral carotid stenosis of 70–99%^14–16^. While other studies suggest that the benefit of such interventions was uncertain^17,18^. A previous study has shown that a stroke-history is correlated with CAS in a Chinese cohort undergoing CABG^7^. However, large-scale clinical studies and meta-analyses analyzing the incidence and risk of CAS in Chinese patients undergoing CABG are still scarce.

The aim of this study was to investigate the contemporary incidence of postoperative stroke and severe CAS in Chinese patients undergoing CABG, to perform a nomogram of severe CAS, and to assess the efficacy of carotid artery stenting in preventing postoperative stroke.

## Methods

### Study design and patients

From 2015 to 2021, 1799 consecutive patients undergoing isolated CABG surgery was recruited at Shandong Provincial Hospital in Jinan, Shandong Province, China. Patients having concomitant cardiovascular surgery (valve replacement, valve repair, and aneurysm removal) were excluded. Anonymous data were retrieved from the electronic medical record system. Ethical approval was obtained from the Biomedical Research Ethic Committee of Shandong Provincial Hospital (SWYX: NO. 2022–490). We confirmed that all research was performed in accordance with local regulations. Informed consent was waived because the study was retrospective according to Biomedical Research Ethic Committee of Shandong Provincial Hospital.

### Postoperative stroke diagnosis

Postoperative stroke was diagnosed by a physician participating in the patient daily care and confirmed by a neurologist and imaging (computed tomography or magnetic resonance imaging). A postoperative stroke was characterized as any new focal or global neurological impairment that was not resolved within 24 h and could not be accounted for by other healthcare procedures.

### Carotid artery stenosis diagnosis

In our institution, 1702 (94.6%) patients were analyzed for the presence and degree of stenosis using bilateral carotid duplex ultrasonography. The peak systolic velocity (PSV) of the internal carotid artery (ICA) and the velocity ratio of the internal and common carotid arteries (ICA: CCA) were recorded in the Duplex measurements. The definition of the degree of stenosis was consistent with radiological guidelines^19,20^, no to mild stenosis (< 50%) defined as PSV < 125 cm/s and ICA:CCA ratio < 2.0, moderate stenosis (50–69%) as PSV of 125 to 229 cm/s or ICA:CCA ratio in the range of 2. 0–3.9, severe stenosis (70–99%) as PSV ≥ 230 cm/s or ICA:CCA ratio ≥ 4.0, carotid artery occlusion was defined as a PSV and ICA:CCA ratio of 0 (Table 1).

**Table 1:** Carotid artery stenosis definition and degree of severity.

Duplex ultrasound (US) measurement	Mild < 50%	Moderate 50–69%	Severe 70–99%
PSVICA (mm/sec)	< 125	125–229	> 230
PSVICA/PSVCCA	< 2	2.0–3.9	> 4.0

*PSVICA*, peak systolic velocity of the internal carotid artery; *PSVCCA*, peak systolic velocity of the common carotid arteries.

### Other diagnosis

Hypertension was defined as repeated measurements of systolic blood pressure (SBP) ≥ 140 mmHg and diastolic blood pressure (DBP) ≥ 90 mmHg or use of anti-hypertensive therapy. type II diabetes mellitus (T2DM) was defined as repeated measurements of episodic plasma glucose values ≥ 200 mg/dl (≥ 11.1 mmol/L), fasting plasma glucose ≥ 126 mg/dl (≥ 7.0 mmol/L) (fasting time 8- 12 h), or use of T2DM prescription drugs. Hyperlipidemia was determined as a serum low-density lipoprotein cholesterol (LDL-C) ≥ 140 mg/dL, high-density lipoprotein cholesterol (HDL-C) ≥ 40 mg/dL, total cholesterol (TCho) level ≥ 220 mg/dL, and serum triglycerides ≥ 150 mg/dL, or the use of lipid-lowering treatments. Chronic kidney disease (CKD) was defined as GFR 30 mg/g for ≥ 3 months.

### CABG and support techniques

The CABG surgical approach (off-pump CABG: median sternotomy or anterior or lateral minimally invasive direct coronary artery bypass [MIDCAB] approach) is determined by the patient cardiac symptoms and the severity of the CAD or CAS. In most cases in this series, the median sternotomy approach is the standard strategy. The MIDCAB approach is preferred for grafting isolated proximal disease of the left anterior descending branch or the first diagonal artery. The operation of carotid artery stenting is determined by the neurologist's assessment and the severity of the CAS (≥ 70%). Percutaneous carotid artery stenting (transfemoral approaches) is performed in most cases one week before CABG. All patients were treated with dual antiplatelet (DAPT) therapy with aspirin and clopidogrel before receiving carotid artery stenting for two days and after the procedure. All carotid artery stent implantation procedures in this study were performed by the same neurosurgeon and their team, using conventional interventional techniques. The patient selection criteria for carotid artery stent implantation in this study were confirmed vascular stenosis of 70% or more by cerebral angiography or clinical symptoms with vascular stenosis of 50% or more.

### End points

Stroke occurring after CABG was the primary study endpoint. Secondary endpoints included in-hospital mortality, postoperative myocardial infarction, length of hospital stay, intubation time, ICU stay, readmission to the ICU, and reoperation.

### Data analysis

#### Presentation

Statistical analyses were performed using SPSS statistical software version 25.0 and Stata statistical software version 16.0. Continuous variables are expressed as mean ± SD or median and interquartile variance (IQR). Categorical data were expressed as frequencies or percentages. The t test or Mann–Whitney U test and χ2 test or Fisher's exact test were employed to test for the presence of significant differences between groups. Values less than 0.05 were considered statistically significant for all tests as two-sided tests.

#### Nomogram

Nomograms are increasingly used for prognostic analysis as a simpler, more intuitive, and more advanced method^21^. Univariate and multivariate logistic regression models were used to explore potential factors and estimate their weights by severe CAS. Variables with *p* < 0.05 and those possible predictor variables in the univariate model were entered into the multivariate logistic regression model and Forward: LR was performed with probabilities of entry and removal of 0.05 and 0.10, respectively. Based on these important risk factors, the screening was candidate nomogram models with appropriate predictive power. The predictive performance of the nomogram and other models to predict severe CAS rates was quantified using the area under the curve (AUC). The capability of the nomogram was also tested by fitting a well-calibrated curve. The clinical utility of nomograms was also carefully investigated using decision curve analysis (DCA) to compensate for the limitation that ROC curves do not achieve optimal sensitivity and specificity simultaneously.

#### Propensity matching

To assess the association between severe CAS and stroke, we used propensity matching to group patients with and without severe CAS. A parsimonious model was first developed using multivariate logistic regression, as described in the previous section. Clinically correlated variables that were not found to be substantially relevant to postoperative stroke were then appended to the fitted model to generate a propensity-matched model. The propensity matching model was quantified using Kdensity plots and propensity matching test plots.

## Results

### Incidence of CAS and postoperative stroke

**Figure 1:**
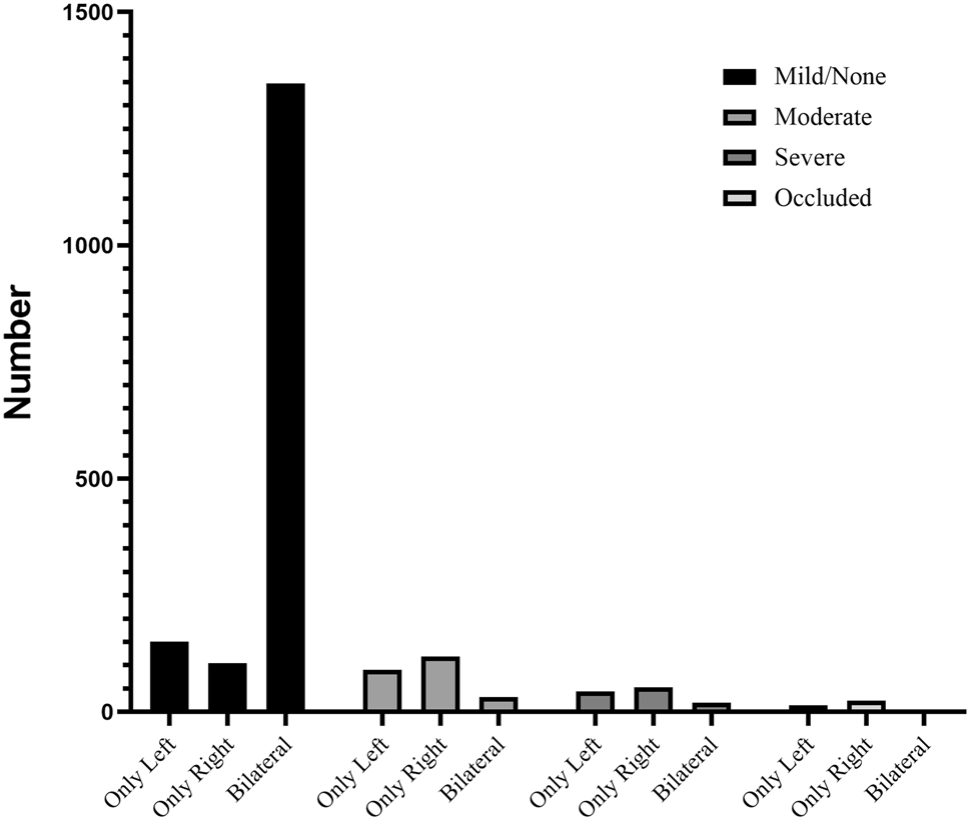
Incidence of carotid artery stenosis. *Note* The incidence of carotid stenosis was classified by the degree of stenosis and unilateral or bilateral.

**Figure 2:**
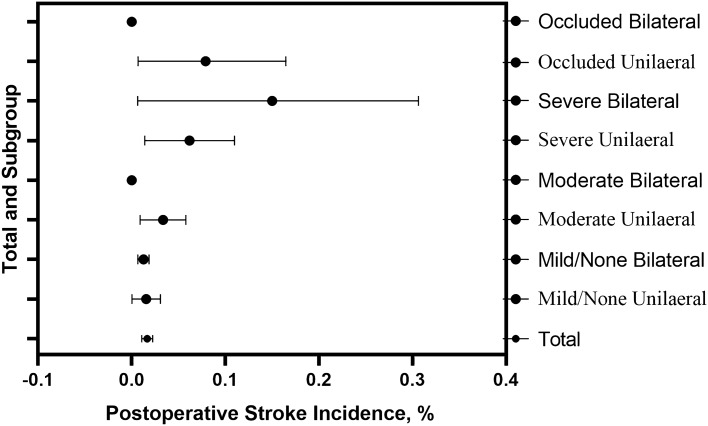
Incidence of postoperative stroke. *Note* The incidence of postoperative stroke was divided according to the degree of carotid stenosis and unilateral or bilateral.

Stroke occurred in 30 (1.67% [95% CI, 1.08%-2.26%]) of 1799 patients. 1702 (94.6%) patients with bilateral carotid duplex ultrasound data were divided into no to mild stenosis, moderate stenosis, severe stenosis, and carotid occlusion groups. Also, carotid stenosis was divided into left-only, right-only, unilateral (left + right-only), and bilateral groups (Table 2, Fig. 1). Patients with stroke had a longer hospital stay (*P* < 0.001) and ICU stay (*P* = 0.001). The optimal cut-off value for age (determined by the ROC curve and Youden index) was 65 years in patients with severe CAS and 60 years in patients with postoperative stroke as a concomitant disease. The clinical characteristics of patients who experienced a stroke were shown in Supplementary Table 1. Stroke occurred in 4 of 256 patients (1.56% [95% CI, 0.04%-3.08%]) in no to mild unilateral groups, 17 of 1347 (1.26% [95% CI, 0.67%- 1.86%]) in no to mild bilateral groups, 7 of 209 (3.35% [95% CI, 0.91%-5.79%]) in moderate unilateral groups, 0 of 30 (0%) in moderate bilateral groups, 6 of 97 (6.19% [95% CI, 1.39%-10.98%]) in severe unilateral groups, 3 of 20 (15.00% [95% CI, 0.65%- 30.65%]) in severe bilateral groups, 3 of 38 (7.89% [95% CI, 0.68%- 16.47%]) in occluded unilateral groups, and 0 of 3 (0%) in occluded bilateral groups (Fig. 2). There was a statistically significant difference in the incidence of postoperative stroke in the severe CAS group (*P* < 0.001), severe unilateral CAS group (*P* = 0.007), severe bilateral CAS group (*P* = 0.001), and occluded unilateral CAS group (*P* = 0.033) compared with all patients. It indicates that the greater the degree of stenosis, the higher the incidence of perioperative stroke, especially in the severe unilateral, severe unilateral, and occluded unilateral groups.

**Table 2:** Incidence of carotid artery stenosis disease (*n* = 1702).

Degree of stenosis	Mild/None	Moderate	Severe	Occluded
Only left	151	90	44	14
Only right	105	119	53	24
Bilateral	1347	32	20	3

### Risk factors for severe CAS

Severe carotid stenosis (70%-99%) was found in 117 of 1702 patients in our patient population (6.87%). Table 3 compared the clinical and demographic characteristics of patients after CABG classified by severe carotid stenosis. In univariate logistic regression analysis, a total of 11 factors (containing age ≥ 65 years, height, SBP, DBP, cerebrovascular accident, CKD, preoperative hemoglobin, C-reactive protein (CRP), B-type natriuretic peptide (BNP), eGFR, and left main disease) were detected statistically associated with the incidence of severe CAS. And multivariate logistic regression analysis found the following independent risk factors for severe CAS: age ≥ 65 years, SBP, DBP, CKD, and preoperative hemoglobin.

**Table 3:** Clinical characteristics of patients after coronary artery bypass grafting classified by severe carotid artery stenosis.

	Severe carotid artery stenosis	None	*P* Value
Patient population (*n*)	117	1585	
Demographic data			
Age (y)	67 (8.00)	64 (10.00)	< 0.001
Age ≥ 60 (*n*)	101 (86.3%)	1090 (68.8%)	< 0.001
Age ≥ 65 (*n*)	78 (66.7%)	742 (46.8%)	< 0.001
Sex, Male (*n*)	77 (65.8%)	1163 (73.4%)	0.076
Height (cm)	163 (11.00)	165 (11.00)	0.002
Weight (kg)	68 (16.00)	70 (15.50)	0.163
BMI (kg/m2)	25.85 (4.72)	25.91 (4.30)	0.684
Medical history			
SBP at Admission (mm Hg)	140 (30.00)	136 (26.50)	0.045
DBP at Admission (mm Hg)	76 (14.50)	79 (16.00)	0.034
MAP (mm Hg)	98.33 (17.33)	98.00 (17.00)	0.923
Hypertension (*n*)	88 (75.2%)	1069 (67.4%)	0.082
Diabetes (*n*)	53 (45.3%)	600 (37.9%)	0.110
Peripheral vascular disease (*n*)	13 (11.1%)	108 (6.8%)	0.081
COPD (*n*)	7 (6.0%)	61 (3.8%)	0.372
History of myocardial infarction (*n*)	51 (43.6%)	669 (42.2%)	0.770
History of cerebrovascular accidents (*n*)	50 (42.7%)	363 (22.9%)	< 0.001
Hyperlipidemia (*n*)	49 (41.9%)	610 (38.5%)	0.467
Chronic kidney disease (*n*)	7 (6.0%)	37 (2.3%)	0.036
History of smoking (*n*)	63 (53.8%)	831 (52.4%)	0.767
Preoperative laboratory tests			
Hemoglobin (g/L)	134.5 (21.00)	141 (20.00)	< 0.001
Platelet (10^9^/L)	214 (89.25)	215 (71.00)	0.710
Lymphocyte (10^9^/L)	1.80 (0.53)	1.70 (0.67)	0.177
Monocyte (10^9^/L)	0.47 (0.24)	0.44 (0.21)	0.257
Neutrophil(10^9^/L)	4.00 (1.69)	3.79 (1.55)	0.116
CRP (mg/L)	2.03 (3.86)	1.47 (2.87)	0.017
BNP (pg/ml)	287.0 (1143.50)	221.7 (489.66)	0.031
PT (s)	11.7 (1.05)	11.6 (1.20)	0.623
PT-INR	1.01 (0.08)	1.01 (0.08)	0.814
Preoperative renal function			
SCr (μmol/L)	72.6 (25.56)	68.3 (18.75)	0.143
eGFR (mL/min/1.73m^2^)	92 (23.00)	96 (17.00)	0.001
Preoperative cardiovascular status			
Left main trunk diseases (*n*)	73 (62.4%)	680 (42.9%)	< 0.001
LVEF (%)	60 (5.00)	60 (5.00)	0.466
Surgical details			
Operation time (h)	4.33 (0.75)	4.33 (0.75)	0.124
Carotid artery stenting (*n*)	24 (20.5%)	4 (0.3%)	< 0.001
OPCABG (*n*)	114 (97.4%)	1546 (97.5%)	1.000
Left atrial appendage closure (*n*)	8 (6.8%)	127 (8.0%)	0.650
Vein graft in-flow from the aorta to target vessels (*n*)	113 (96.6%)	1537 (97.0%)	1.000
MIDCAB (*n*)	1 (0.9%)	15 (0.9%)	1.000
Postoperative laboratory tests			
Hemoglobin (g/L)	107 (22.00)	116 (21.00)	0.001
Platelet (10^9^/L)	174 (67.50)	178 (67.00)	0.988
Lymphocyte(10^9^/L)	0.76 (0.41)	0.74 (0.43)	0.524
Monocyte(10^9^/L)	0.78 (0.38)	0.75 (0.43)	0.360
Neutrophil (10^9^/L)	10.63 (4.38)	11.56 (4.29)	0.024
Outcomes			
Stroke (*n*)	9 (7.7%)	21 (1.3%)	< 0.001
In-hospital mortality (*n*)	1 (0.9%)	5 (0.3%)	0.887
Postoperative myocardial infarction (*n*)	0 (0%)	4 (0.3%)	1.000
Postoperative Af (*n*)	21 (17.9%)	254 (16.0%)	0.585
Anticoagulation therapy for postoperative Af(*n*)	13 (11.1%)	163 (10.3%)	0.777
Hospital stays (d)	17.76 (8.19)	17.53 (7.27)	0.545
Intubation time (h)	11.00 (4.59)	11.17 (3.90)	0.428
ICU Stays (d)	2.08 (1.24)	2.06 (1.07)	0.581
Re-Admission to ICU (*n*)	1 (0.9%)	7 (0.4%)	1.000
Reoperation (*n*)	0 (0%)	6 (0.4%)	1.000

*BMI*: Body Mass Index; *SBP*: Systolic Blood Pressure; *DBP*: Diastolic Blood Pressure; *MAP*: Mean Arterial Pressure; *COPD*: Chronic Obstructive Pulmonary Disease; *CRP*: C-Reactive Protein; *BNP*: B-Type Natriuretic Peptide; *PT*: Prothrombin Time; *INR*: International Normalized Ratio; *SCr*: Serum Creatinine; *eGFR*: Estimated Glomerular Filtration Rate; *LVEF*: Left Ventricular Ejection Fraction; *MIDCAB*: Minimally Invasive Direct Coronary Artery Bypass; *OPCABG*: off-pump Coronary Artery Bypass grafting; *ICU*: Intensive Care Unit; Af, Atrial Fibrillation.

The categorical variables in the table are presented by the number of cases (with percentage) and the continuous variables are expressed by the median (with interquartile range) or mean (with standard deviation). *P* Value: Compare the patients with and without severe carotid artery stenosis. *P* values were the results of unpaired *t*-test or Mann–Whitney U test for continuous variables, and χ2 test or Fisher’s exact test for categorical variables.

### Nomogram model for severe CAS

**Figure 3:**
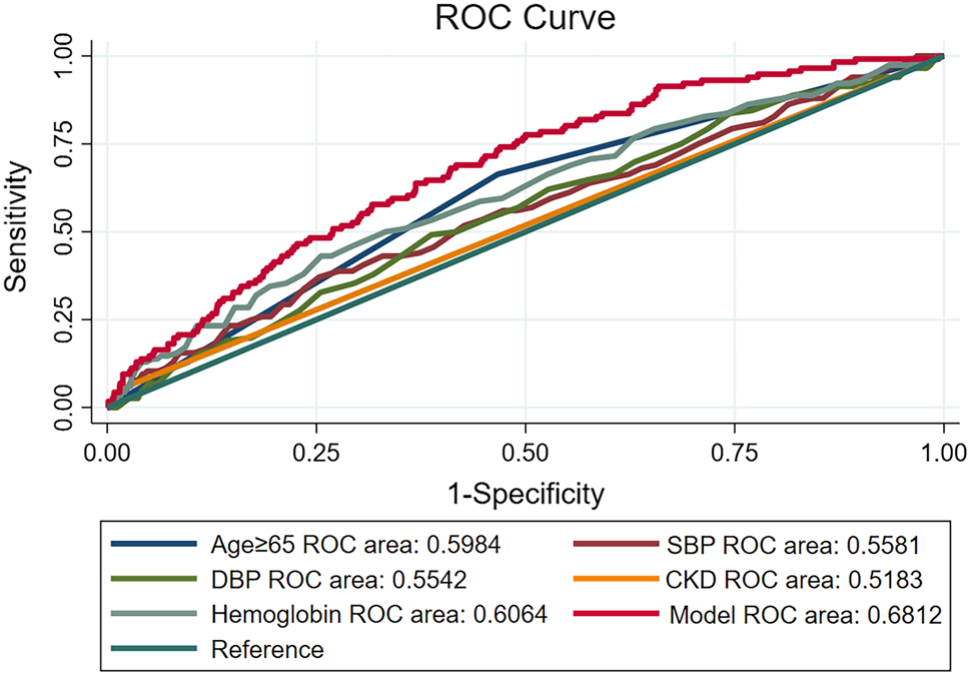
ROC curves for the nomogram model constructed from age ≥ 65 years, SBP, DBP, CKD, and hemoglobin. *Note* ROC curves of the nomogram model with age ≥ 65 years, SBP, DBP, CKD, and hemoglobin. ROC, receiver operating characteristic; SBP, systolic blood pressure; DBP, diastolic blood pressure; CKD, chronic kidney disease.

**Figure 4:**
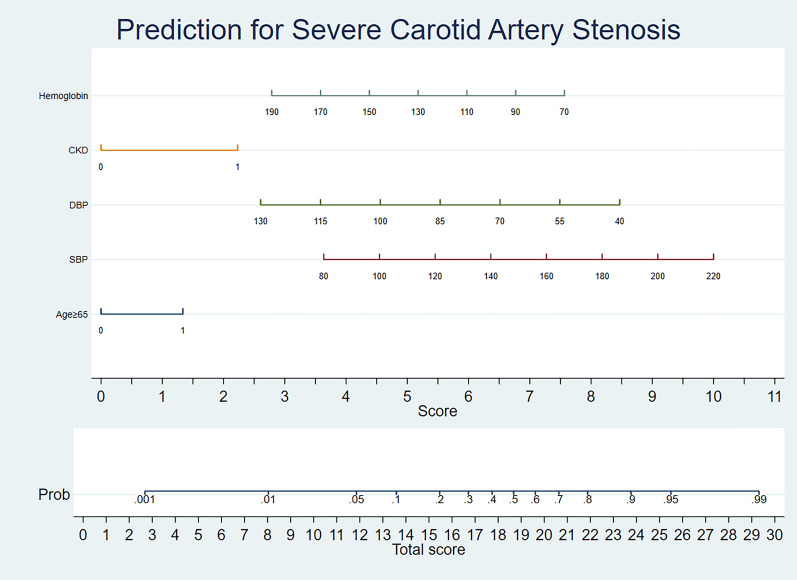
Nomogram for predicting severe carotid stenosis. *Note* The scores of each variable were summed to obtain the total score and a vertical line was drawn on the total score to obtain the corresponding probability of death.

**Figure 5:**
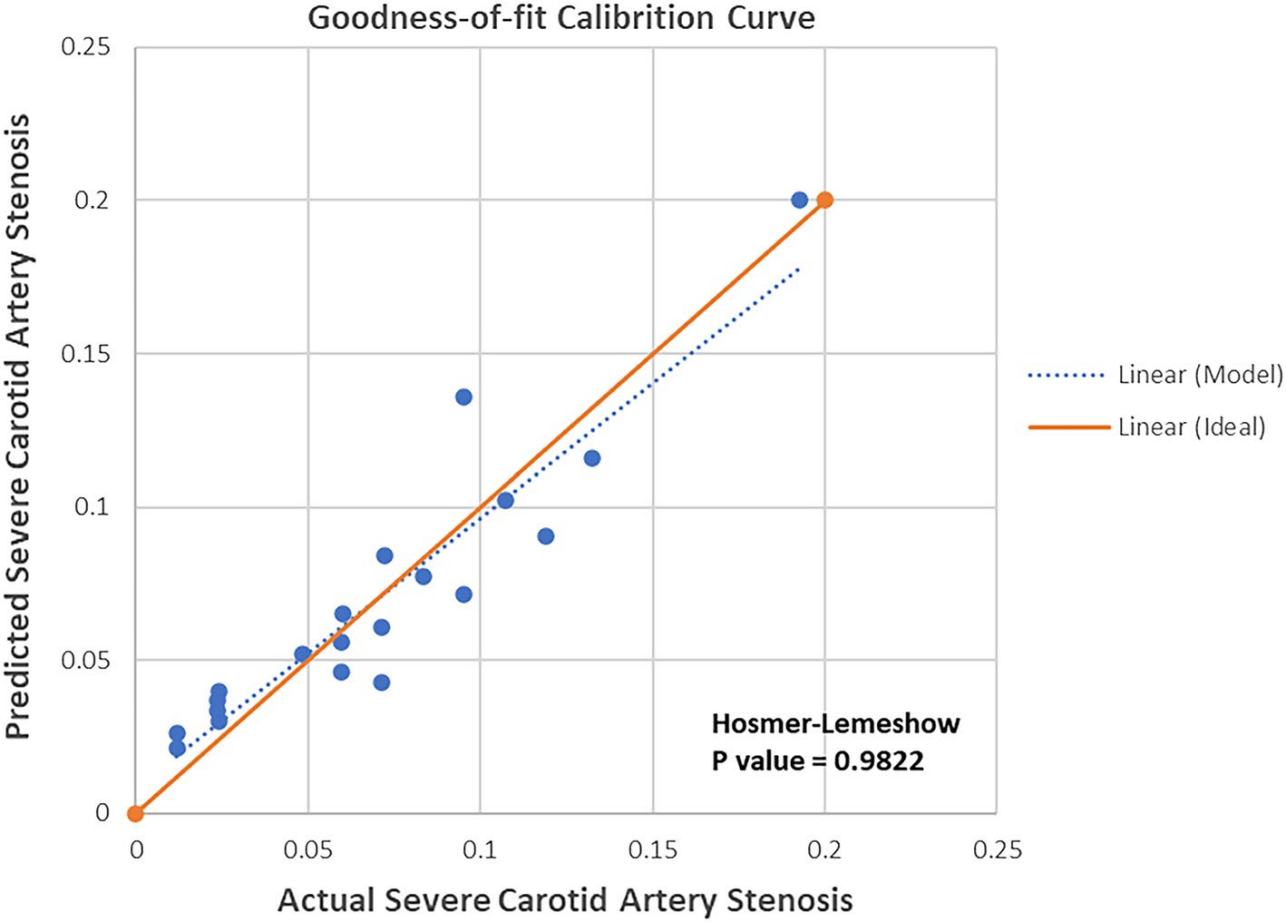
Calibration curve for predicting severe carotid stenosis by nomogram. *Note* Fitted calibration curve for predictive nomograms (Hosmer–Lemeshow *P* value = 0.9822).

**Figure 6:**
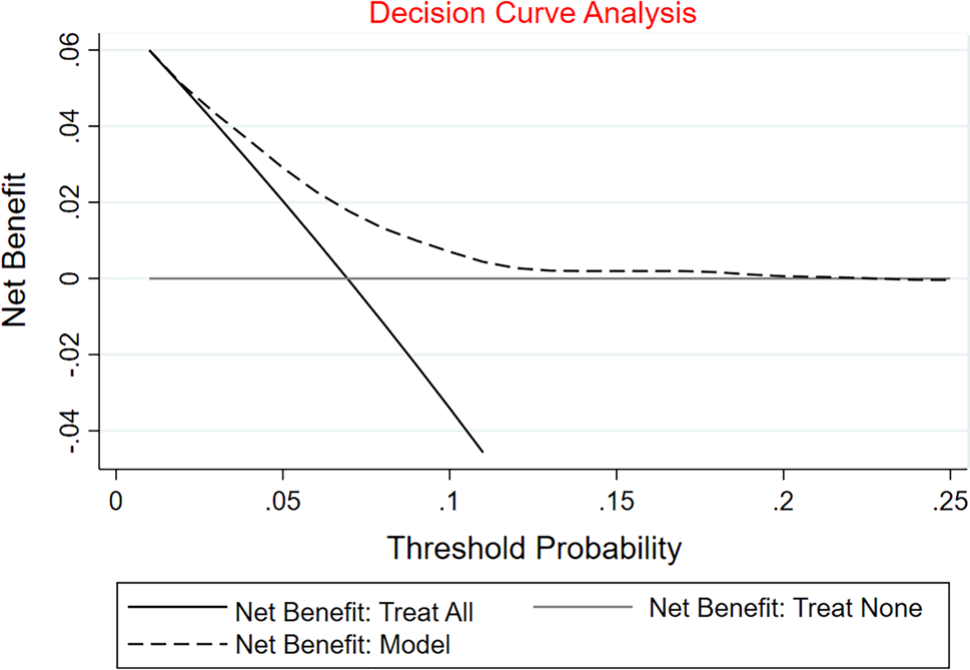
DCA curves of the predictive nomogram model constructed from age ≥ 65 years, SBP, DBP, CKD, and hemoglobin. *Note* The horizontal axis represents the threshold value, which is the reference probability of whether a patient receives treatment, and the vertical axis represents the net benefit rate after subtracting the disadvantage from the advantage. A larger net benefit for the same threshold probability means that the patient receives the greatest benefit using the model's diagnosis. the closer the curve in the DCA plot is to the top, the higher the value of the model's diagnosis. DCA, decision curve analysis; SBP, systolic blood pressure; DBP, diastolic blood pressure; CKD, chronic kidney disease.

To develop an optimal nomogram model, we evaluated the individual and combined performance of these five factors using ROC analysis (Fig. 3). The individual AUCs for age ≥ 65 years, SBP, DBP, CKD, preoperative hemoglobin, and prediction model were 0.598, 0.558, 0.554, 0.518, 0.606, and 0.681, respectively. In addition, as seen in Fig. 4, each of the chosen biomarkers was assigned a proportional score based on its value on the nomogram. To confirm the generality of the nomogram in predicting the incidence of severe CAS, we screened the nomogram for comprehensive validation. The well-fitted calibration curves showed high agreement in predicting the incidence of severe CAS, as shown in Fig. 5 (Hosmer–Lemeshow P value = 0.982). Also, DCA curves were created in Fig. 6. Regardless of the threshold, the nomogram model performed well across the various predictors, which ensured that maximum clinical benefit was achieved. Overall, the DCA curves indicate that the nomogram model is feasible to make a valuable and favorable assessment.

### Propensity matching

**Figure 7:**
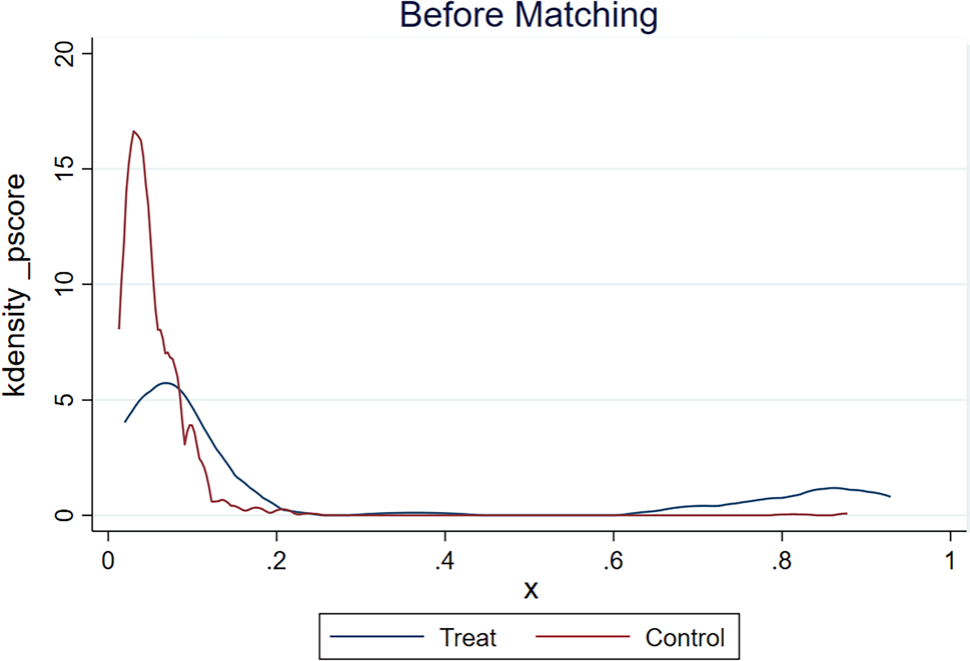
Kdensity plots of the propensity-matched model for severe carotid stenosis.*Note* Figure 7 shows the Kdensity before matching.

**Figure 8:**
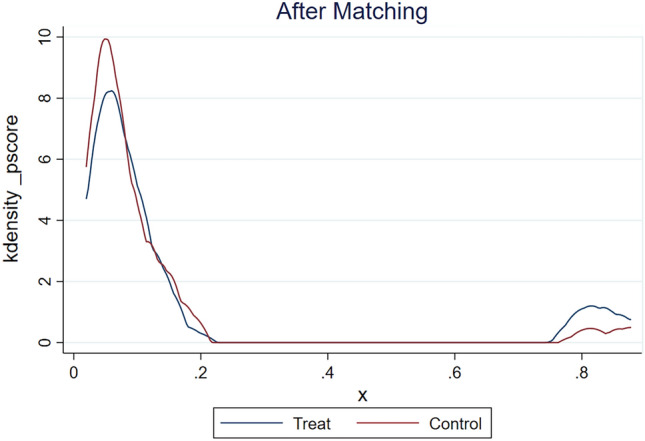
Kdensity plots of the propensity-matched model for severe carotid stenosis. *Note* Figure 8 shows the Kdensity after matching.

**Figure 9:**
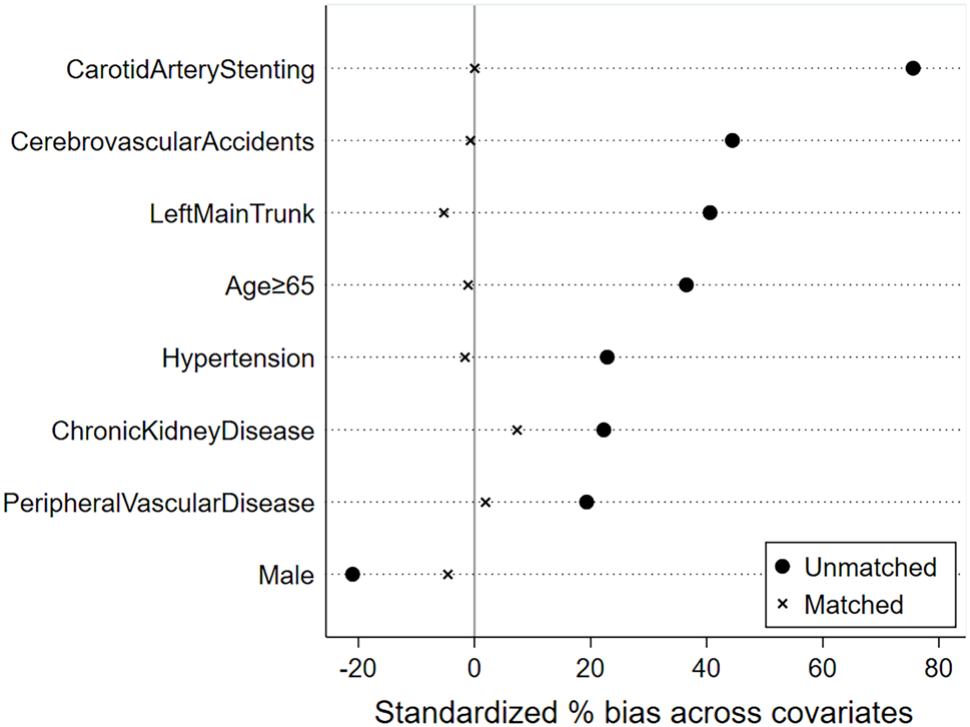
Propensity-matched test plots for the severe carotid stenosis model. *Note* Propensity-matched test plots for the severe carotid stenosis model.

To assess the association between severe CAS and postoperative stroke, we used a propensity-matched approach to group patients with and without severe CAS. A total of 8 factors (including age ≥ 65 years, male, hypertension, peripheral vascular disease, cerebrovascular accident, chronic kidney disease, left main artery disease, and carotid stenting) were included to form a propensity-matched model. The propensity matching model was quantified using Kdensity plots (Fig. 7 shows the Kdensity before matching and Fig. 8 shows the Kdensity after matching) and propensity matching test plots (Fig. 9). These tests showed that the two groups after propensity matching achieved a good match. The clinical and demographic characteristics of the patients after propensity-matched analysis by severe CAS were shown in Table 4. In univariate logistic regression analysis, a statistically significant difference in ICU length of stay (*p* = 0.041) was found. After matching, stroke occurred in 6 of 75 patients in the severe CAS group (8.0% [95% CI, 1.86%-14.14%]) and in 0 of 75 patients in the non-severe CAS group (0%) (*p* = 0.028).

**Table 4:** Clinical characteristics of patients after propensity matching analysis.

	Severe carotid artery stenosis	None	*P* Value
Patient population (*n*)	75	75	
Demographic data			
Age (y)	67 (9.00)	65 (8.00)	0.046
Age ≥ 60 (*n*)	64 (85.3%)	56 (74.7%)	0.102
Age ≥ 65 (*n*)	49 (65.3%)	38 (50.7%)	0.069
Sex, Male (*n*)	51 (68.0%)	51 (68.0%)	1.000
Height (cm)	163.5 (12.00)	165 (12.00)	0.281
Weight (kg)	68 (17.00)	69 (14.00)	0.260
BMI (kg/m2)	25.31 (4.56)	26.07 (4.05)	0.348
Medical history			
SBP at Admission (mm Hg)	138 (29.00)	139 (27.00)	0.656
DBP at Admission (mm Hg)	75 (16.00)	80 (14.00)	0.128
MAP (mm Hg)	98.33 (21.33)	99.67 (14.00)	0.500
Hypertension (*n*)	57 (76.0%)	58 (77.3%)	0.847
Diabetes (*n*)	34 (45.3%)	42 (56.0%)	0.191
Peripheral vascular disease (*n*)	8 (10.7%)	8 (10.7%)	1.000
COPD (*n*)	3 (4.0%)	4 (5.3%)	1.000
History of myocardial infarction (*n*)	29 (38.7%)	37 (49.3%)	0.188
History of cerebrovascular accidents (*n*)	31 (41.3%)	28 (37.3%)	0.616
Hyperlipidemia (*n*)	29 (38.7%)	32 (42.7%)	0.618
Chronic kidney disease (*n*)	3 (4.0%)	5 (6.7%)	0.719
History of smoking (*n*)	42 (56.0%)	38 (50.7%)	0.513
Preoperative laboratory tests			
Hemoglobin (g/L)	134 (22.00)	138 (20.50)	0.234
Platelet (10^9^/L)	212 (85.00)	228.5 (80.75)	0.475
Lymphocyte (10^9^/L)	1.79 (0.53)	1.75 (0.81)	0.977
Monocyte (10^9^/L)	0.48 (0.26)	0.45 (0.23)	0.406
Neutrophil(10^9^/L)	3.79 (1.96)	4.27 (1.62)	0.165
CRP (mg/L)	1.71 (3.63)	1.18 (3.52)	0.251
BNP (pg/ml)	219.9 (636.15)	243.55 (456.33)	0.657
PT (s)	11.70 (1.18)	11.55 (1.18)	0.987
PT-INR	1.01 (0.07)	1.02 (0.07)	0.511
Preoperative renal function			
SCr (μmol/L)	72.8 (24.91)	71.0 (24.1)	0.493
eGFR (mL/min/1.73m^2^)	92 (23.75)	98 (15.00)	0.129
Preoperative cardiovascular status			
Left main trunk diseases (*n*)	47 (62.7%)	47 (62.7%)	1.000
LVEF (%)	60 (4.00)	60 (7.00)	0.664
Surgical details			
Operation time (h)	4.25 (0.70)	4.25 (0.82)	0.680
Carotid artery stenting (*n*)	10 (13.3%)	4 (5.3%)	0.092
OPCABG (*n*)	72 (96.0%)	73 (97.3%)	1.000
Left atrial appendage closure (*n*)	5 (6.7%)	6 (8.0%)	0.754
Vein graft in-flow from the aorta to target vessels (*n*)	73 (97.3%)	74 (98.7%)	1.000
MIDCAB (*n*)	0 (0%)	0 (0%)	1.000
Postoperative laboratory tests			
Hemoglobin (g/L)	107 (21.50)	111 (31.00)	0.509
Platelet (10^9^/L)	176 (67.50)	172 (93.00)	0.616
Lymphocyte(10^9^/L)	0.76 (0.45)	0.74 (0.53)	0.863
Monocyte(10^9^/L)	0.83 (0.43)	0.74 (0.29)	0.238
Neutrophil (10^9^/L)	11.00 (4.81)	11.85 (3.08)	0.137
Outcomes			
Stroke (*n*)	6 (8.0%)	0 (0%)	0.028
In-hospital mortality (*n*)	0 (0%)	1 (1.3%)	1.000
Postoperative myocardial infarction (*n*)	0 (0%)	0 (0%)	1.000
Postoperative Af (*n*)	13 (17.3%)	12 (16.0%)	0.827
Anticoagulation therapy for postoperative Af(*n*)	8 (10.7%)	8 (10.7%)	1.000
Hospital stays (d)	17.79 (9.62)	19.33 (8.12)	0.975
Intubation time (h)	11.83 (3.42)	11.00 (4.68)	0.851
ICU stays (d)	1.97 (1.09)	2.87 (1.99)	0.041
Re-admission to ICU (*n*)	1 (1.3%)	0 (0%)	1.000
Reoperation (*n*)	0 (0%)	0 (0%)	1.000

*BMI*: Body Mass Index; *SBP*: Systolic Blood Pressure; *DBP*: Diastolic Blood Pressure; *MAP*: Mean Arterial Pressure; *COPD*: Chronic Obstructive Pulmonary Disease; *CRP*: C-Reactive Protein; *BNP*: B-Type Natriuretic Peptide; *PT*: Prothrombin Time; *INR*: International Normalized Ratio; *SCr*: Serum Creatinine; *eGFR*: Estimated Glomerular Filtration Rate; LVEF: Left Ventricular Ejection Fraction; *MIDCAB*: Minimally Invasive Direct Coronary Artery Bypass; *OPCABG*: off-pump Coronary Artery Bypass grafting; *ICU*: Intensive Care Unit; Af, Atrial Fibrillation The categorical variables in the table are presented by the number of cases (with percentage) and the continuous variables are expressed by the median (with interquartile range) or mean (with standard deviation). *P* Value: Compare the patients with and without severe carotid artery stenosis. *P* values were the results of unpaired t-test or Mann–Whitney *U* test for continuous variables, and χ2 test or Fisher’s exact test for categorical variables.

### Evaluating of carotid artery stenting

The symptomatic status of 13 patients with severe CAS was shown in Table 5 and 11 received carotid artery stenting. The severe CAS group was divided into a carotid stenting subgroup and a non-carotid stenting subgroup, with stroke occurring in 1 of 24 patients in the carotid stenting subgroup (4.17% [95% CI, -3.83%-12.16%]) and in 8 of 93 patients in the no-carotid stenting subgroup (8.60% [95% CI, 2.90%-14.30%]) (*p* = 0.766). Similarly, stroke occurred in 1 of 29 patients in the carotid stenting subgroup (3.45% [95% CI, 3.31%-10.21%]) and in 29 of 1673 patients in the no carotid stenting subgroup (1.73% [95% CI, 1.11%-2.36%]) among all patients (*P* = 1.000).

**Table 5:** The Symptomatic status of patients with severe carotid artery stenosis.

Total	13 (11.2%)
Dizziness	5 (4.3%)
Amaurosis	2 (1.7%)
Limb numbness	2 (1.7%)
Aphasia	1 (0.9%)
Hemiplegia	2 (1.7%)
Sensory impairment	1 (0.9%)

## Discussion

Stroke has continued to be one of the potentially destructive complications of CABG, with significant clinical and economic implications for patients and healthcare systems^6,22,23^. In previous studies, incidence of stroke was 3.0%^24^, 1.7%^25^, 1.6%^6^ among patients undergoing CABG mainly in American and European area. Over the past 40 years, the incidence of stroke has declined despite increasing patient risk profiles, which benefited from improvements in preoperative assessment, surgical techniques, and postoperative care. The incidence and risk of CAS in Chinese patients undergoing CABG is still unclear. Thus, we compiled 1799 consecutive patients who underwent isolated CABG surgery at Shandong Provincial Hospital. We observed a similar incidence of postoperative stroke (1.67%) in our Chinese CABG cohort. Patients with stroke after CABG had a longer hospital stay and longer time in the ICU than patients without stroke. However, there was no statistical difference in the in-hospital survival between the two groups.

It is believed that carotid stenosis is one of the risk factors for stroke after CABG^5,25,26^. In addition, a previous meta-analysis of US and European CAS studies showed that the perioperative risk of stroke after cardiac surgery varied from 3.8% to 7.4% for patients with ≥ 50% CAS and increased to 2% to 9.1% for patients with ≥ 70% CAS^27^. In our study, the incidence of postoperative stroke after CABG was found to be similar, with 3.7% of patients with ≥ 50% CAS and 7.7% of patients with ≥ 70% CAS. A higher incidence of postoperative stroke was found in the severe unilateral group (*P* = 0.007), the severe bilateral group (*P* = 0.001), and the occluded unilateral group (*P* = 0.033) compared to the overall patients. It was shown that a more severe stenosis was associated with a higher incidence of postoperative stroke, particularly in the severe unilateral, severe unilateral, and occluded unilateral groups. After propensity matching, the incidence of stroke was 8.0% in the severe CAS group and 0% in the non-severe CAS group (*p* = 0.028). The present study is consistent with the prevailing view that severe CAS is an independent risk factor for postoperative stroke.

The incidences of significant CAS stenosis (≥ 50%) were 12.8% to 22.1% and severe CAS stenosis (≥ 70%) were 4.6% to 5.0% in patients with CABG in the United States and European countries^20,28^. In our Chinese CABG cohort, the incidence of CAS with stenosis ≥ 50% and ≥ 70% was 19.2% and 6.9%, which were consistent with those of previous studies.

An optimal predictive nomogram incorporating age ≥ 65 years, SBP, DBP, CKD, and preoperative hemoglobin to predict severe CAS in patients with CABG was successfully established and carefully evaluated. The available evidence suggests that the nomogram could effectively predict patient prognosis, and its simplicity and intuitive nature facilitate the interpretation by clinical staff^29,30^. To our knowledge, the present study is the first report on the development of a nomogram for predicting severe CAS in patients with CABG using a number of baseline tests. In a multivariate logistic regression model, we observed that age ≥ 65 years, SBP, DBP, CKD, and preoperative hemoglobin were all independently associated with the incidence of severe CAS. Satisfactory accuracy was observed when the above five variables were included in the nomogram model (AUC = 0.681). In recent years, many studies have reported that the incidence of CAS increases with age^31,32^. In Durand DJ's study, age over 65 years was shown to be a significant predictor of CAS^33^. In previously conducted studies, SBP or DBP was considered as a predictor of carotid stenosis^32,34,35^. In addition, Puz P et al. found that CKD was an independent risk factor for symptomatic internal CAS^36^. It could be interpreted that CKD is independently associated with carotid atherosclerosis^37^. Furthermore, Dijk JM and other colleagues demonstrated that increased hemoglobin levels were associated with reduced severity of atherosclerosis, assessed as the presence of ≥ 50% CAS^38^. Fortunately, our findings showed that age ≥ 65 years, SBP, DBP, CKD, and hemoglobin were also significantly associated with the incidence of severe CAS in patients undergoing CABG, in agreement with these published findings. To avoid the limitations of a single predictor and to obtain higher prediction accuracy, this study combined five tested predictors to form a nomogram model. Our data confirmed that the nomogram was more effective at predicting severe CAS than any single predictor (AUC = 0.681). Moreover, DCA curves have been commonly used in many studies to assess the efficacy of specific clinical approaches^39,40^. In this study, we also used DCA curves to examine the underlying clinical effects of the nomogram, and our findings suggest that the nomogram was more valuable than other indicators in predicting the incidence of severe CAS.

Carotid endarterectomy is considered to be an effective treatment for both symptomatic patients and asymptomatic patients with CAS^41,42^. Carotid artery stenting is another treatment technique. However, evidence for the effect of carotid stenting in patients with severe CAS undergoing CABG is lacking, and its effectiveness preventing postoperative stroke remains controversial^43^. Although there were 117 patients with severe carotid artery stenosis confirmed by carotid ultrasound in the study, the number of patients who ultimately underwent carotid artery stenting did not exceed 30. It was because the degree of stenosis confirmed by cerebral angiography did not meet the above criteria in some of the remaining patients. And other patients could not tolerate carotid artery surgery or combined surgery due to severe conditions (such as frequent angina attacks or severe heart failure). Our study suggested that carotid stenting is not effective in preventing postoperative stroke. Possible explanations are as follows. First, carotid stenosis may be a marker of high atherosclerotic burden and stroke risk, rather than a direct stroke mechanism in most patients. Second, there was relatively few data on carotid artery stenting in our study. Third, combined CABG and carotid stenting are at higher risk than CABG alone.

The study has several limitations. First, the study design was retrospective and non-randomized. In addition, data from a single medical center were analyzed and only selective patients were included. Due to less than thirty patients who underwent carotid artery stent implantation in this study, sample size was indeed limited. Therefore, the results of the study may not be generalizable to other Asian populations and studies with larger sample sizes and higher-quality are needed to confirm our findings. Second, we were unable to determine whether the etiology of each stroke was embolic, thrombotic, or hypoperfused. In this study, all stroke patients were diagnosed with ischemic stroke. However, due to the clinical situations in China and the difficulty in differential diagnosis of stroke etiology, it is challenging to accurately determine whether the stroke is caused by thrombosis, embolism, plaque rupture, or perioperative hypoperfusion and etc. In theory, we could differentiate the etiology of stroke to some extent based on the course of the disease and cranial magnetic resonance imaging (MRI). However, in practice, it is difficult to perform these assessments. Especially since most patients have steel wires fixating the sternum in the thoracic cavity postoperatively, and many patients have unstable conditions in the early stage after surgery, which makes it challenging to perform comprehensive cranial MRI examinations. Third, some patients who underwent emergency surgery were not included in the study because of the lack of ultrasound. Finally, current guidelines give priority to surgical treatment of CAS before CABG, especially in symptomatic patients^44^. Carotid stenting is an alternative treatment and in this study patients with CAS requiring carotid artery treatment were treated only endovascularly. We hope to continue further research comparing carotid endarterectomy and stenting in patients undergoing CABG in the future.

## Conclusions

The incidence of postoperative stroke was 1.67% and severe CAS was in 6.87% patients who underwent CABG in the last 7 years in a single center in China, similar to recent studies in the United States and Europe. More severe carotid stenosis was found to be associated with a higher incidence of postoperative stroke, especially in the severe unilateral group, severe unilateral group, and occluded unilateral group, and severe CAS was an independent risk factor for postoperative stroke. A nomogram constructed from age ≥ 65 years, SBP, DBP, CKD, and hemoglobin could provide an accurate and favorable prediction of the incidence of severe CAS. Carotid artery stenting is ineffective in preventing postoperative stroke.

### Supplementary Information

Supplementary Table 1.

## Data Availability

The data that support the findings of this study are available from the corresponding author upon reasonable request.
