# Supplementary material for: The role of carotid artery stenosis in predicting stroke after coronary artery bypass grafting in a Chinese cohort study
Source: Sci Rep. 2023 Dec 6;13:21536. doi: 10.1038/s41598-023-47640-5 (PMC10700536; doi:10.1038/s41598-023-47640-5)
Supplement: Supplementary file 1 — Supplementary Table 1. [file 41598_2023_47640_MOESM1_ESM.docx]

| **Supplementary Table 1 Clinical Characteristics of Patients after Coronary Artery Bypass Grafting Classified by Stroke** | | | |
| --- | --- | --- | --- |
|  | **Stroke** | **None** | **P Value** |
| **Patient Population (n)** | 30 | 1672 |  |
| **Demographic Data** |  | | |
| Age (y) | 66 (7.25) | 64 (10.00) | 0.257 |
| Age≥60 (n) | 24 (80.0%) | 1167 (69.8%) | 0.227 |
| Sex, Male (n) | 24 (80.0%) | 1216 (72.7%) | 0.375 |
| Height (cm) | 169 (8.00) | 165 (11.00) | 0.047 |
| Weight (kg) | 74.5 (11.25) | 70 (16.00) | 0.070 |
| BMI (kg/m2) | 26.95 (3.94) | 25.91 (4.31) | 0.223 |
| **Medical History** |  | | |
| SBP at Admission (mm Hg) | 144 (20.25) | 136 (26.00) | 0.010 |
| DBP at Admission (mm Hg) | 79.5 (17.75) | 79 (15.00) | 0.438 |
| MAP (mm Hg) | 102.33 (19.92) | 98.00 (16.67) | 0.103 |
| Hypertension (n) | 23 (76.7%) | 1134 (67.8%) | 0.303 |
| Diabetes (n) | 16 (53.3%) | 637 (38.1%) | 0.089 |
| Peripheral Vascular Disease (n) | 1 (3.3%) | 120 (7.2%) | 0.650 |
| COPD (n) | 3 (10.0%) | 65 (3.9%) | 0.221 |
| History of Myocardial Infarction (n) | 17 (56.7%) | 703 (42.0%) | 0.108 |
| History of Cerebrovascular Accidents (n) | 15 (50.0%) | 398 (23.8%) | 0.001 |
| Hyperlipidemia (n) | 12 (40.0%) | 647 (38.7%) | 0.884 |
| Chronic Kidney Disease (n) | 1 (3.3%) | 43 (2.6%) | 1.000 |
| History of Smoking (n) | 17 (56.7%) | 878 (52.5%) | 0.651 |
| Severe Carotid Artery Stenosis (n) | 9 (30.0%) | 108 (6.5%) | <0.001 |
| **Preoperative Laboratory Tests** |  | | |
| Hemoglobin (g/L) | 137.5 (16.50) | 140 (20.00) | 0.171 |
| Platelet (10^9^/L) | 236 (100.00) | 215 (71.00) | 0.346 |
| Lymphocyte (10^9^/L) | 1.69 (0.72) | 1.72 (0.67) | 0.774 |
| Monocyte (10^9^/L) | 0.50 (0.27) | 0.44 (0.21) | 0.097 |
| Neutrophil(10^9^/L) | 4.04 (2.28) | 3.80 (1.56) | 0.291 |
| CRP (mg/L) | 3.28 (5.87) | 1.49 (2.86) | 0.088 |
| BNP (pg/ml) | 398.5 (866.25) | 222.4 (502.44) | 0.188 |
| PT (s) | 11.35 (1.13) | 11.6 (1.20) | 0.075 |
| PT-INR | 0.99 (0.07) | 1.01 (0.08) | 0.043 |
| **Preoperative Renal Function** |  | | |
| SCr (μmol/L) | 66.0 (24.90) | 68.7 (19.50) | 0.761 |
| eGFR (mL/min/1.73m^2^) | 93.5 (23.50) | 96 (18.00) | 0.508 |
| **Preoperative Cardiovascular Status** |  | | |
| Left Main Trunk Diseases (n) | 18 (60.0%) | 736 (44.0%) | 0.081 |
| LVEF (%) | 58.5 (5.25) | 60 (5.00) | 0.394 |
| **Surgical Details** |  | | |
| Operation Time (h) | 4.50 (0.77) | 4.33 (0.78) | 0.103 |
| Carotid Artery Stenting (n) | 1 (3.3%) | 27 (1.6%) | 0.993 |
| MIDCAB (n) | 0 (0%) | 16 (1.0%) | 1.000 |
| **Postoperative Laboratory Tests** |  | | |
| Hemoglobin (g/L) | 110.5 (18.00) | 115 (20.00) | 0.600 |
| Platelet (10^9^/L) | 183.5 (62.00) | 177 (67.00) | 0.578 |
| Lymphocyte(10^9^/L) | 0.77 (0.47) | 0.75 (0.43) | 0.700 |
| Monocyte(10^9^/L) | 0.89 (0.47) | 0.76 (0.43) | 0.140 |
| Neutrophil (10^9^/L) | 11.85 (4.82) | 11.46 (4.31) | 0.670 |
| **Outcomes** |  | | |
| In-Hospital Mortality (n) | 1 (3.3%) | 5 (0.3%) | 0.220 |
| Postoperative Myocardial Infarction (n) | 0 (0%) | 4 (0.2%) | 1.000 |
| Hospital Stays (d) | 22.53 (14.19) | 17.48 (7.24) | <0.001 |
| Intubation Time (h) | 11.54 (5.13) | 11.17 (3.91) | 0.116 |
| ICU Stays (d) | 2.93 (2.44) | 2.04 (1.07) | 0.001 |
| Re-Admission to ICU (n) | 1 (3.3%) | 7 (0.4%) | 0.334 |
| Reoperation (n) | 1 (3.3%) | 5 (0.3%) | 0.220 |
| Abbreviations: BMI: Body Mass Index; SBP: Systolic Blood Pressure; DBP: Diastolic Blood Pressure; MAP: Mean Arterial Pressure; COPD: Chronic Obstructive Pulmonary Disease; CRP: C-Reactive Protein;  BNP: B-Type Natriuretic Peptide; PT: Prothrombin Time; INR: International Normalized Ratio; SCr: Serum Creatinine; eGFR: Estimated Glomerular Filtration Rate; LVEF: Left Ventricular Ejection Fraction;  MIDCAB: Minimally Invasive Direct Coronary Artery Bypass; ICU: Intensive Care Unit. NOTE. The categorical variables in the table are presented by the number of cases (with percentage) and the continuous variables are expressed by the median (with interquartile range) or mean (with standard deviation). P Value: Compare the patients with and without severe carotid artery stenosis. P values were the results of unpaired t-test or Mann-Whitney U test for continuous variables, and χ2 test or Fisher’s exact test for categorical variables. | | | |
